# Advancing iris melanoma brachytherapy: Eye plaque models for Monte Carlo simulations and 3D dosimetric datasets

**DOI:** 10.1002/mp.70439

**Published:** 2026-05-12

**Authors:** Marwa Djedouani, Alex Viner, Elizabeth M. Fletcher, Rowan M. Thomson

**Affiliations:** ^1^ Carleton Laboratory for Radiotherapy Physics, Department of Physics Carleton University Ottawa Ontario Canada

**Keywords:** dose calculation, eye plaque brachytherapy, iris melanoma, Monte Carlo

## Abstract

**Purpose:**

To develop and benchmark eye plaque models for Monte Carlo (MC) simulation of iris melanoma brachytherapy and to develop a database of 3D dose distributions for the iris plaques containing 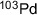, 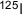, and 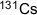 seeds.

**Acquisition and Validation Methods:**

Five iris plaque models are developed with egs_brachy using published dimensions and material data; previously benchmarked seed models are used. Three plaque models are based on those used by the Mayo Clinic, consisting of a modification of the COMS 22 mm plaque design with a 10 mm void in the center surrounded by an inner collimating lip; plaques span $180^{\circ }$, $270^{\circ }$, and $360^{\circ }$ arcs. Two additional plaques are modeled: a modified Iris‐270 plaque with no collimating lips; a partially‐loaded COMS 22 mm plaque with no insert. Plaques are simulated in a water phantom with dose scored in (0.05 ${\mathrm{cm})}^{3}$ voxels. Simulations under TG‐43 conditions are also carried out. Doses are compared to previously published data for validation.

**Data Format and Usage Notes:**

The eye plaque models will be distributed with egs_brachy on GitHub (https://github.com/clrp‐code/egs_brachy), along with an input file to facilitate custom simulations. The dosimetric database (https://doi.org/10.5281/zenodo.14776641) is comprised of 3D dose distributions for each plaque type, simulation condition, and radionuclides.

**Potential applications:**

The iris plaque models enable custom simulations with the open‐access egs_brachy code. The database of 3D dose distributions supports advanced dose evaluations, as recommended by AAPM Task Group 221 on Ocular Brachytherapy. Overall, this work supports adoption of model‐based dose evaluations for brachytherapy as recommended by TG‐186.

## 1 INTRODUCTION

Uveal melanoma is one of the most prevalent forms of intraocular cancer, with 3%–4% of cases involving the iris.^1^ Treatments for iris melanoma include surgical resection, enucleation, and eye plaque brachytherapy.^2^ Plaque brachytherapy of uveal melanoma offers similar tumor control to enucleation but with eye preservation.^3^ The small size of the eye and steep dose gradients mean that accurate dosimetry for eye plaque brachytherapy is critical for treatment planning and understanding treatment outcomes. Traditionally, eye plaque therapy dose calculations for photon‐emitting sources follow the American Association of Physicists in Medicine (AAPM) Task Group No. 43 (TG‐43) protocol.^4^ TG‐43 dose calculations neglect inhomogeneities in the vicinity of the seeds, including the plaque backing and insert. This has led to considerable dose differences observed for dose evaluations that account for the effect of the plaque backing and insert compared with TG‐43 calculations: for example, dose decreases of 40% in the tumor and 90% in organs at risk have been reported for 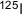 and 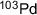 seeds in the standardized plaques of the Collaborative Ocular Melanoma Study (COMS).^5^, ^6^, ^7^, ^8^ For treatment of iris melanoma, dose differences of 30% at critical points of interest have been observed in comparing TG‐43 calculations to those accounting for the plaque backing and insert.^9^

Model‐based dose calculation algorithms (MBDCAs), such as Monte Carlo (MC) simulations, differ from the TG‐43 formalism by providing more accurate dose evaluations that account for patient‐ and treatment‐specific scatter conditions and heterogeneities. TG‐129 recommends considering the plaque in dose evaluations;^10^ TG‐221 recommends carrying out a MBDCA dose calculation in parallel with TG‐43 to accurately calculate patient dose.^2^ Similarly, the joint AAPM‐ESTRO‐ABS‐ABG TG‐186 report recommends the clinical adoption of MBDCAs and that MBCDA calculations be performed in parallel to the standard TG‐43 calculations.^11^

This work uses egs_brachy, a freely‐distributed and open‐source EGSnrc application, to develop five eye plaque models based on those used for iris melanoma brachytherapy and calculate 3D dose distributions. The plaques that we consider were previously modeled with BrachyDose and limited dosimetric results were published by Thomson et al,^9^ however, neither BrachyDose nor those plaque models were ever released. This dataset article describes the geometric models developed in egs_brachy of the plaques, presents 3D dose distributions from simulations with egs_brachy, and compares results to those previously published.^9^ It is hoped that the freely‐available benchmarked plaque models that will be distributed with egs_brachy and the 3D dose distributions developed in this work will support the advancement of iris melanoma treatments.

## 2 ACQUISITION AND VALIDATION METHODS

### 2.1 Monte Carlo simulations of eye plaques

MC simulations are performed using the EGSnrc^12^ application egs_brachy ^13^ (GitHub commit hash 0219903, 2023 version). Comprehensive benchmarking of egs_brachy for brachytherapy (radionuclide) seeds and eye plaques can be found in previous publications.^13^, ^14^, ^15^, ^16^, ^17^, ^18^ The EGSnrc^12^ default transport parameters are generally used, following the low‐energy default parameters distributed with egs_brachy.^19^ Electron transport is not simulated for the current work (although electron transport may be modeled in egs_brachy ^13^). The photon energy transport cutoff is set to 1 keV. Rayleigh scattering, photoelectric absorption, Compton scattering, and fluorescent emission of characteristic X rays are simulated. Photon cross‐sections are from the XCOM database.^20^ Dose is approximated as collision kerma scored with a track length estimator using mass energy absorption coefficients distributed with egs_brachy (application g as distributed with the EGSnrc package before 2017^19^). Recent improvements^21^ in the g application show that the mass energy absorption coefficient values would change by up to a maximum of 0.2% using the updated release of EGSnrc. There is ambiguity in whether renormalized or unrenormalized Scofield photoelectric cross sections are in better agreement with experimental data;^22^ the current work utilizes “unrenormalized” photoelectric cross sections, consistent with EGSnrc default parameters.^19^

**FIGURE 1:**
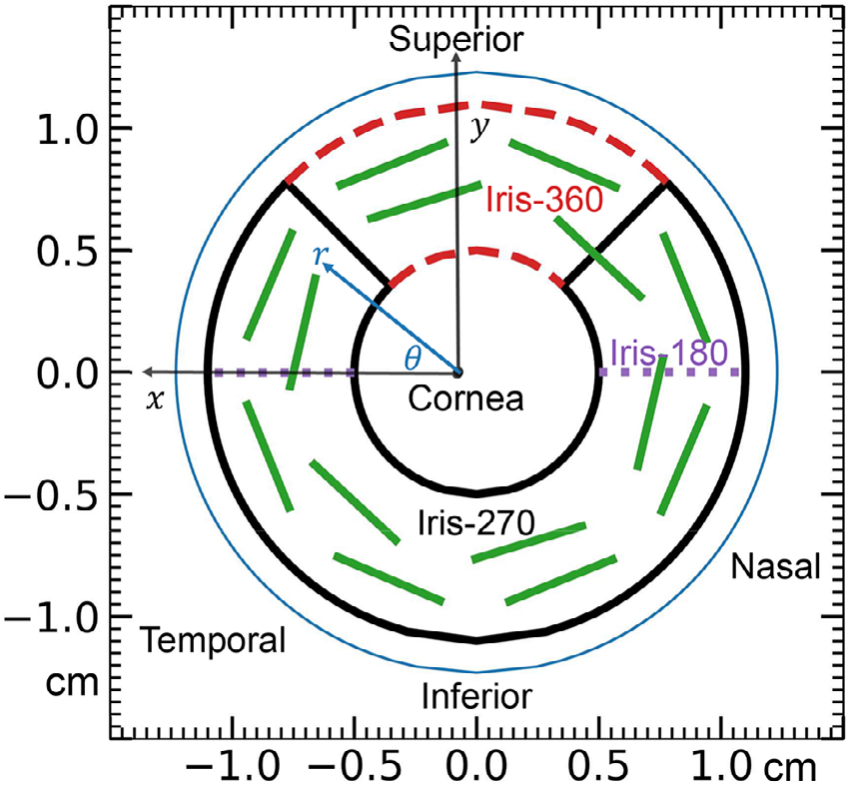
Schematic depiction of the iris plaques on a right eye, viewed from the front (inspired by Ref. 9). The x,y axes in the plaque coordinate system are shown, as well as the associated r,θ polar coordinates used to describe seed positions in Table 2. The green bars depict seed locations; all 14 are present for Iris‐360, whereas the Iris‐180 and Iris‐270 contain subsets of 6 and 10 seeds, respectively, that are wholly contained within the plaque outlines in the diagram.

The egs++ class library geometry module is used to develop the eye plaque models using data from the previous publication on modeling with BrachyDose.^9^ The five plaque models developed in the present work are summarized in Table 1. The plaque models conform to an idealized eye which is a sphere of radius of 1.23 cm with a sclera 0.1 cm thick. In the following, two coordinate systems are used, as follows.^7^, ^10^, ^23^ For the plaque coordinate system (x,y,z), the plaque's central axis (CAX) defines the z‐axis, with z = 0 at the inner sclera (at the eye anterior); the x and y axes are transverse to the plaque. The x and y axis in the plaque coordinate system are visible in Figure 1 as are the associated polar coordinates (r,θ) which are used to identify seed center positions. The eye reference frame (X,Y,Z) defines the origin at the center of the eye with a vertical Z‐axis and horizontal X and Y axes. Focusing on a right eye, X increases towards the anterior of the eye, Y towards the nasal side, and Z in the cranial direction. The two coordinate systems are related through x=−Y, y=Z, and z=1.13cm−X.

**TABLE 1:** Overview of plaques simulated with name, backing, insert material, and number of seeds (configuration given in Table 2).

Name	Backing	Insert	# of seeds (config.)
Iris‐180	modified COMS; central void; $180^{\circ }$ span	Silastic	8 (Iris‐180)
Iris‐270	modified COMS; central void; $270^{\circ }$ span	Silastic	10 (Iris‐270)
Iris‐360	modified COMS; central void; $360^{\circ }$ span	Silastic	14 (Iris‐360)
Iris‐270‐No‐Lips	Iris‐270 with no collimating lips	Water	10 (Iris‐270)
COMS‐No‐Insert	standard 22 mm COMS	Water	10 (Iris‐270)

For the iris plaques,^9^ the backing and collimating lips are made of the gold‐alloy Modulay with elemental composition by mass 77% Au, 14% Ag, 8% Cu and 1% Pd and mass density ρ = 15.8 g/${\mathrm{cm}}^{3}$; the seed carrier is Silastic (silicone polymer): 39.9% Si, 28.9% O, 24.9% C, 6.3% H, 0.005% Pt; ρ = 1.12 g/${\mathrm{cm}}^{3}$.^10^, ^15^ As reported by Thomson et al,^9^ the plaques are similar to the 22 mm COMS plaque with a Modulay shell that is 0.5 mm thick with an outer radius of curvature of 15.05 mm. The collimating lips are 2.7 mm in length and 0.5 mm thick. Unlike the COMS plaques, the iris plaques have a 10 mm diameter void in the center of the plaque (where water is modeled) surrounded by a 0.5 mm thick Modulay segment.^9^ The plaques span three different arc lengths; $180^{\circ }$ (Iris‐180), $270^{\circ }$ (Iris‐270) and $360^{\circ }$ (Iris‐360) (Figure 1). Both the Iris‐180 and Iris‐270 have a 0.5 mm thick Modulay segment connecting the outer and inner collimating lips. All iris plaques have a Silastic seed carrier insert which holds seeds in configurations given in Table 2.

**TABLE 2:** Seed center coordinates specified in the plaque coordinate system.

	xy‐plane		Iris		
z (cm)	r (cm)	θ (degrees)	360	270	180
0.115	0.92	23	✓	✓	
		68	✓		
		113	✓		
		158	✓	✓	
		203	✓	✓	✓
		248	✓	✓	✓
		293	✓	✓	✓
		338	✓	✓	✓
−0.029	0.73	13	✓	✓	
		73	✓		
		133	✓		
		193	✓	✓	
		253	✓	✓	✓
		313	✓	✓	✓

The other two plaque models simulated are based on plaques reported elsewhere for treatment of iris melanoma.^24^, ^25^, ^26^ The “Iris‐270‐No‐Lips” plaque is a modified version of the Iris‐270 plaque with no collimating lips and no Silastic insert (with water modeled instead).^24^, ^25^ The “COMS‐No‐Insert” plaque is a partially‐loaded 22 mm COMS plaque backing that does not contain a Silastic insert.^26^ Seed positions are the same as for the Iris‐270 plaque (Table 2). For both the Iris‐270‐No‐Lips and COMS‐No‐Insert plaques, water is modeled where there is no Silastic seed carrier; the thin layer of fixative that is used in treatments to hold the seeds is not modeled. The approach of modeling water where there is no Silastic seed carrier is consistent with the prior work of Thomson et al^9^ and is thus needed for verification. Future work could consider the effects of having another medium in this space, including air or eye secretions in a realistic non‐water patient model.

Dose is scored in a (2.55 ${\mathrm{cm})}^{3}$ subvolume made of (0.05 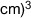 voxels in the center of a (30 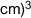 water phantom (ρ = 0.998 g/${\mathrm{cm}}^{3}$) which extends from ‐15 cm ≤x,y,z≤ 15 cm. The egs_brachy voxel volume correction^13^, ^15^ option (with $10^{8}$ random points/${\mathrm{cm}}^{3}$) is used for voxels that overlap with plaque components: dose is only scored in the part of the voxel that is not occupied by the plaque.

Simulations of the plaques containing 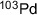, 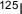, or 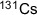 seeds are carried out. The seed models are 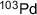 Theragenics TheraSeed model 200,^15^, ^27^ 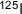 Amersham OncoSeed model 6711^15^, ^28^ and 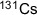 Isoray model CS‐1 Rev2.^15^, ^29^ The egs_brachy models of the seeds were developed and benchmarked as part of the initial egs_brachy paper;^13^ they were also included in the 2020 CLRP TG43v2 database.^15^ Consistent with the CLRP TG43v2 database, photons are initialized in seeds with spectra from the NCRP report 58^30^ (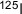) and the NNDC spectra^31^ (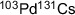). The mean energy of photons emitted from the seeds is 27.34 keV for 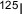, 20.51 keV for 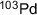 and 30.29 keV for 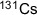 from previous egs_brachy calculations.^15^ The particle recycling and phase space source features of egs_brachy are not used.

In addition to simulations with the plaque at the center of the water phantom, “TG43‐sim” simulations are carried out that mimic TG‐43 assumptions.^7^, ^9^, ^32^ These simulations use the same water phantom and the same seed positions but there is no plaque backing or insert material present. Furthermore, egs_brachy's “superposition” run mode is used so that no interseed effects are modeled.

Each simulation models $6.5\times 10^{10}$ histories. History‐by‐history statistics are used to estimate (type A) statistical uncertainties in egs_brachy.^13^ In this work, statistical uncertainties are less than 0.22% on the plaque central axis, 0.04% on $D_{\mathrm{Rx}}$ (dose at the prescription point) and 0.19% at the macula corresponding to the largest uncertainty of the six points of interest in the eye considered (see Table 3). These Type A uncertainties are generally smaller than those for other sources of uncertainty (Type B). Uncertainties in cross section data for the low energy of the 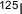 source are often estimated at 2%;^7^, ^29^, ^32^, ^33^ recent work by Valdez‐Cortez et al^34^ suggest uncertainties of around 1.5% in mass‐energy absorption coefficients are dominant in the uncertainty analysis for low‐energy MC simulations (due to uncertainty in photoelectric cross sections). An estimate of 2% is assumed for the uncertainty on seed geometry,^32^ accounting for differences between individually‐manufactured seeds and internal motion of seed components. The uncertainty on the source energy spectrum is taken as 0.1%.^33^

**TABLE 3:** Coordinates of points of interest in the eye coordinate system (X,Y,Z) with each point's distance from the eye center indicated (R).

	Coordinate (cm)			
Location	X	Y	Z	R
Cornea	1.13	0.00	0.00	1.13
Sclera	0.90	0.00	−0.69	1.13
Lens	0.77	0.00	0.00	0.77
Eye center	0.00	0.00	0.00	0.00
Macula	−1.13	0.00	0.00	1.13
Optic disk	−1.06	0.40	0.00	1.13
Rx point	0.74	0.00	−0.57	0.93

Simulations with egs_brachy determine values of dose per history (in each voxel) which are scaled by dividing by the air kerma strength per history ($S_{K}^{\text{hist}}$; previously calculated for the NIST WAFAC detector geometry as part of the CLRP TG43v2 database^15^) to obtain values of dose per unit seed air kerma strength (Gy 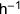 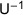 where 1 U=1 cGy ${\mathrm{cm}}^{2}$ 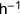). The $S_{K}^{\text{hist}}$ values are $3.7666\left(7\right)\times 10^{-14}$ Gy ${\mathrm{cm}}^{2}$/hist (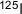 model 6711), $6.4261\left(6\right)\times 10^{-14}$ Gy ${\mathrm{cm}}^{2}$/hist (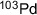 TheraSeed model 200) and $3.7155\left(3\right)\times 10^{-14}$ Gy ${\mathrm{cm}}^{2}$/hist (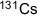 model CS‐1) (Ref. [15], https://physics.carleton.ca/clrp/egs_brachy/seed_database_v2).

### 2.2 Dose comparisons

**FIGURE 2:**
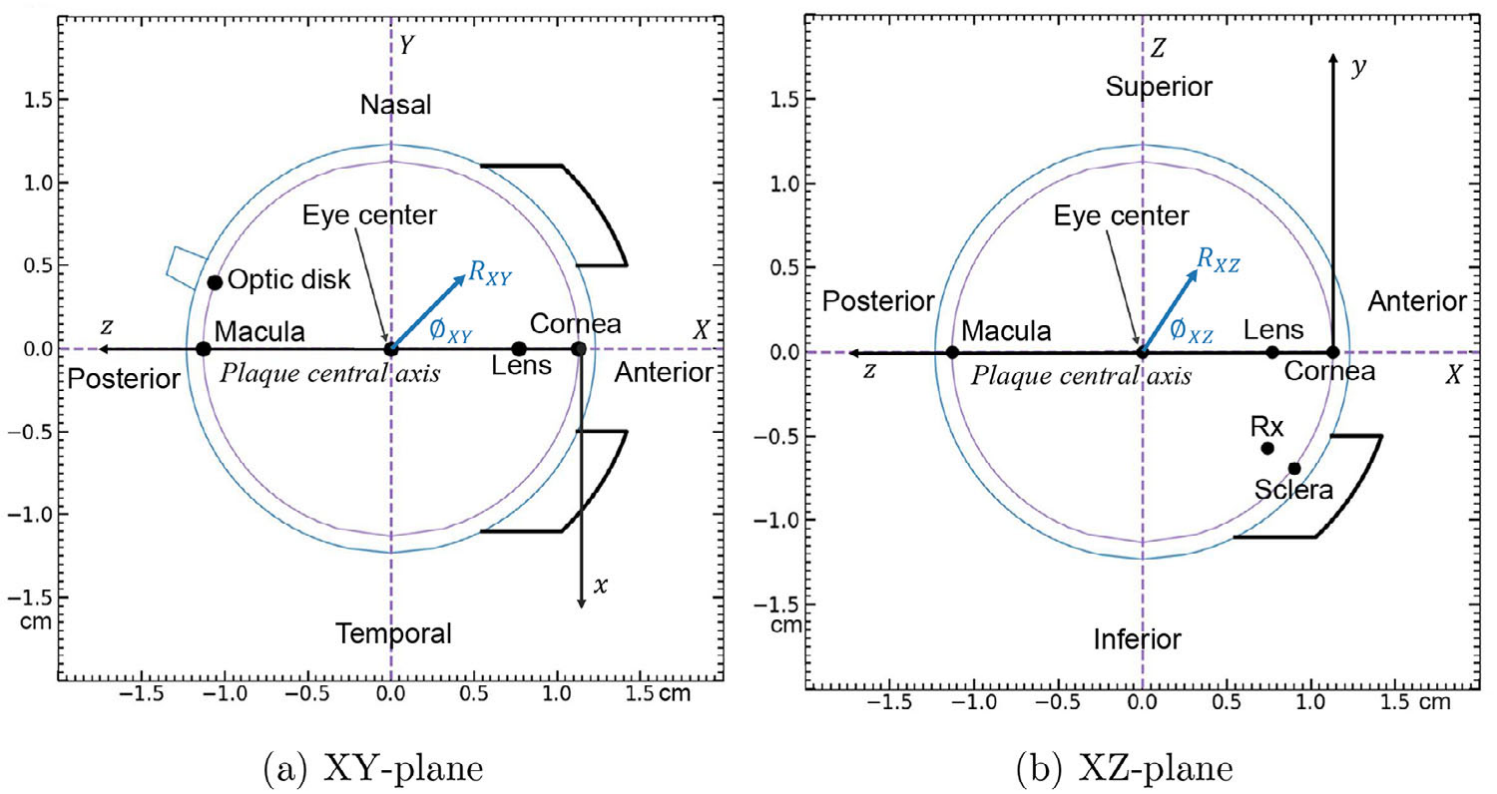
Schematic diagram depicting points of interest for an Iris‐270 plaque placed upon a right eye for the (a) XY‐plane and (b) XZ‐plane. Plaque (x,y,z) and center of eye (X,Y,Z) coordinate systems are shown.

For validation, egs_brachy doses for each plaque are compared to previously published BrachyDose data.^9^ In the following, doses are plotted along the plaque's central axis (z), at fixed radii in both the XY and XZ planes, and at six points of interest in the eye (cornea, sclera, lens, eye center, macula, and optic disk) – see Figure 2. The coordinates of the points of interest and the prescription point (Rx) in the eye coordinate system (X,Y,Z) are given in Table 3. The prescription point is 2 mm towards the eye center from the inner sclera.^9^ For data validation, doses are presented in two different ways in the present section: as a percentage of TG43‐sim doses and as a percentage of the dose to the prescription dose ($D_{Rx}$). This enables comparison with the results of Thomson et al.^9^

**FIGURE 3:**
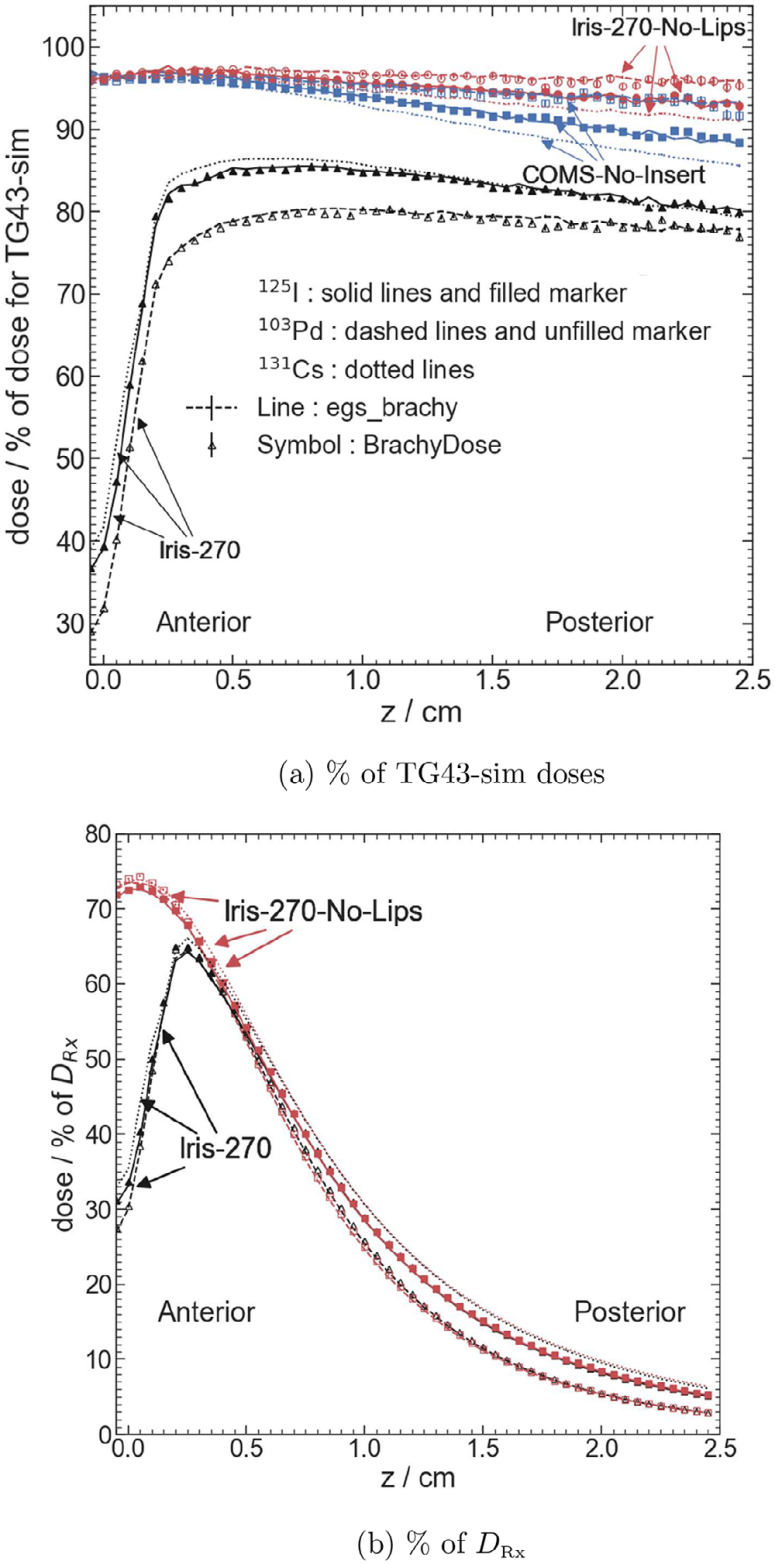
Comparison of doses along the central axis as a percentage of (a) TG43‐sim doses and (b) $D_{\mathrm{Rx}}$ for egs_brachy (lines) and BrachyDose (symbols) for Iris‐270, Iris‐270‐No‐Lips, and COMS‐No‐Insert plaques for 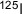 (solid lines, filled markers) 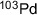 (dashed lines, unfilled markers) and 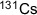 (dotted lines; egs_brachy results only) seeds. Error bars, representing statistical uncertainties, are often too small to see.

Overall, the egs_brachy dose distributions are in agreement with the BrachyDose results that were previously published.^9^ Figure 3a shows dose as a percentage of TG43‐sim doses along the central axis for Iris‐270, Iris‐270‐No‐Lips, and COMS‐No‐Insert plaques for both 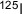 and 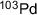 seeds. Differences between egs_brachy and BrachyDose results are small, observed to be at most 1.3% for 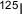, and 1.6% for 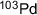. The largest dose differences across all plaques and radionuclides are observed for the Iris‐270 plaque, with an average dose difference of 0.7% for 0.05 cm ≤z≤ 0.2 cm.

Similarly, Figure 3b presents egs_brachy dose distributions along the central axis as a percentage of $D_{\mathrm{Rx}}$ for Iris‐270 and Iris‐270‐No‐Lips plaques for both 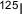 and 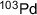 seeds. Dose differences between egs_brachy and BrachyDose are less than 1.9% (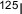) and 1.2% (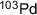). The largest discrepancies are observed for the Iris‐270 plaque near the void at its center at 0.1 cm ≤z≤ 0.2 cm.

Furthermore, Figure 3a and b include egs_brachy dose distributions along the central axis for 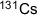 seeds. No comparable results for plaques using 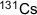 seeds were ever published. The mean energies for 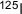 (27.34 keV) and 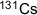 (30.29 keV) result in overall similar dose distributions. Subsequent dose comparisons will focus on 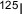 and 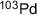 simulations.

**FIGURE 4:**
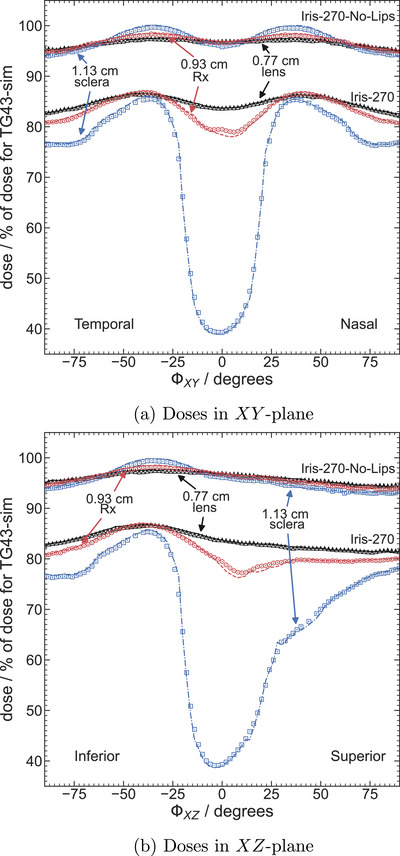
Comparison of doses as a percent of TG43‐sim doses versus angle for egs_brachy (lines) and BrachyDose (symbols) at fixed radii in the (a) XY‐plane and (b) XZ‐plane for Iris‐270 and Iris‐270‐No‐Lips with 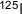 seeds.

Dose distributions for egs_brachy (as a percentage of TG43‐sim doses) versus angle at fixed radii in the XY plane are shown in Figure 4a. Results are presented for Iris‐270 and Iris‐270‐No‐Lips plaques with 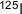 seeds at radii of 0.77 cm (lens), 0.93 cm (Rx), and 1.13 cm (sclera) and compared to BrachyDose results. Percent differences between the egs_brachy and BrachyDose results are less than 0.54% for the fixed radius of 0.77 cm, 1.1% for 0.93 cm, and 2.9% for 1.13 cm. The largest disparities are found at the fixed radius of 1.13 cm for the Iris‐270 plaque, for $-22^{\circ }\leq \phi_{XY}\leq -14^{\circ }$, where doses differ by 1%. Further comparisons are shown in Figure 4b where doses at fixed radii in the XZ plane are shown for Iris‐270 and Iris‐270‐No‐Lips plaques containing 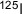 seeds. In general, there is agreement between egs_brachy and BrachyDose results with differences of at most 0.49% for 0.77 cm, 1.2% for 0.93 cm, and 3.6% for 1.13 cm. The largest differences are found within $-2^{\circ }\leq \phi \leq 10^{\circ }$ for the fixed radius of 0.93 cm for the Iris‐270 plaque and for the fixed radius of 1.13 cm for the Iris‐270‐No‐Lips plaque.

**FIGURE 5:**
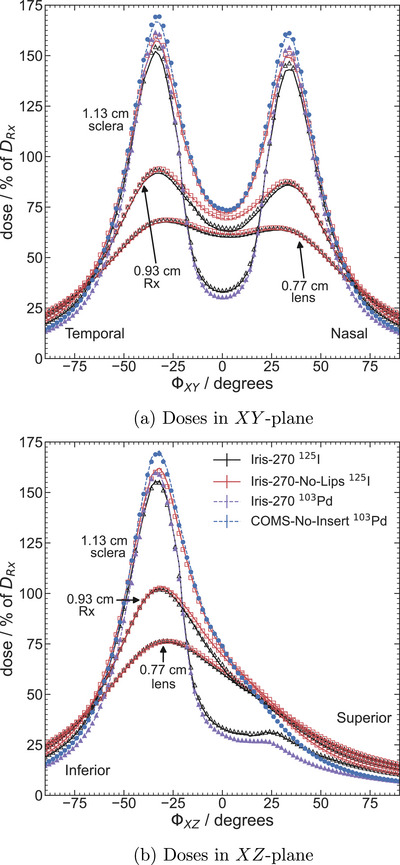
Comparison of doses as a percent of $D_{\mathrm{Rx}}$ versus angle for egs_brachy (lines) and BrachyDose (symbols) at fixed radii in the (a) XY plane and (b) XZ plane for the following plaques and nuclides; Iris‐270, Iris‐270‐No‐Lips for 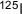 seeds (solid lines, filled markers) and Iris‐270 and COMS‐No‐Insert for 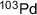 seeds (dashed lines, unfilled markers).

Similarly to Figure 4a, Figure 5a shows the dose distributions of egs_brachy as a percentage of $D_{\mathrm{Rx}}$ at fixed radii in the XY plane. The Iris‐270 and Iris‐270‐No‐Lips plaques are depicted for 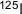 seeds at 0.77 cm (lens), 0.93 cm (Rx) and 1.13 cm (sclera). Additionally, results for the Iris‐270 and COMS‐No‐Insert plaques for 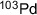 are presented at a fixed radius of 1.13 cm. Comparison to BrachyDose doses shows a difference of at most 1.8% at the fixed radius of 0.77 cm, 2.2% for 0.93 cm and 4.0% for 1.13 cm. The largest variations across all plaques are observed at the fixed radius of 1.13 cm within $-38^{\circ }\leq \phi \leq -20^{\circ }$ for Iris‐270 containing either 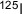 and 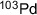, with an average difference of approximately 2.9%. Similar figures of doses at fixed radii in the XZ plane can be seen in Figure 5b. Percent differences between results for both codes are less than 0.89% for a fixed radii of 0.77 cm, 1.8% for 0.93 cm and 7.1% for 1.13 cm.

Doses at six points of interest in the eye (cornea, sclera, lens, eye center, macula, and optic disk) for egs_brachy are presented in Table 4 as a percent of TG43‐sim doses for all modeled plaques, along with BrachyDose values from Thomson et al.^9^ Agreement of egs_brachy results with those from BrachyDose is observed with both radionuclides; the largest differences are 0.9% for 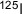 and 0.7% for 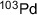. Across all plaques, the greatest discrepancies are observed for the COMS‐No‐Insert plaque with 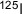 seeds and Iris‐180 with 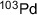 seeds; an average difference of 0.4% is observed for both plaques. Similarly, Table 5 presents doses as a percentage of $D_{\mathrm{Rx}}$ for all modeled plaques and both radionuclides. Comparison to BrachyDose shows agreement with a difference of at most 0.9% for 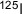 and 1.3% for 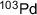. The largest variation across all plaques was observed for Iris‐360, with an average difference of about 0.4% for 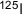 and 0.5% for 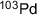.

**TABLE 4:** Comparison of doses as a percent of TG43‐sim doses for egs_brachy ($D_{eb}$) and BrachyDose ($D_{BD}$) at points of interest. Statistical uncertainties are less than 0.6% for BrachyDose ^9^ and 0.23% for egs_brachy.

		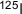		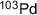	
Plaques	Points of interest	$D_{BD}$	$D_{eb}$	$D_{BD}$	$D_{eb}$
	Cornea	39.3	39.1	31.9	31.6
	Sclera	85.2	85.8	75.2	75.7
	Lens	83.7	83.7	76.8	76.9
Iris‐270	Eye center	84.8	84.8	80.2	80.1
	Macula	81.1	80.7	78.2	78.0
	Optic disk	81.2	80.6	77.2	77.5
	Rx	86.6	87.1	78.7	79.1
	Cornea	96.1	96.2	96.4	96.6
	Sclera	99.4	99.9	98.7	99.2
	Lens	96.9	96.9	97.2	97.1
Iris‐270‐No‐Lips	Eye center	95.6	95.5	96.7	96.7
	Macula	93.6	93.3	96.1	95.8
	Optic disk	94.0	93.4	96.2	95.8
	Rx	98.1	98.6	97.8	98.2
	Cornea	96.5	96.9	95.8	96.5
	Sclera	99.4	99.8	98.4	98.8
	Lens	96.2	96.4	96.2	96.4
COMS‐No‐Insert	Eye center	93.4	93.4	95.0	95.2
	Macula	89.6	88.7	93.0	93.2
	Optic disk	88.4	88.9	93.7	93.6
	Rx	97.7	98.3	97.3	97.7
	Cornea	37.4	37.2	30.2	30.0
	Sclera	83.8	84.3	74.3	74.9
	Lens	83.5	83.7	76.7	76.9
Iris‐360	Eye center	84.5	84.7	80.0	80.1
	Macula	80.8	80.8	78.3	77.8
	Optic disk	80.9	80.8	76.9	77.2
	Rx	85.8	86.2	78.0	78.4
	Cornea	44.2	43.6	36.0	35.5
	Sclera	86.4	86.9	76.2	76.7
	Lens	83.7	84.0	76.9	77.2
Iris‐180	Eye center	84.7	85.1	79.9	80.3
	Macula	80.7	81.1	77.1	77.7
	Optic disk	81.0	81.1	77.2	77.5
	Rx	88.0	88.2	79.8	80.0

**TABLE 5:** Comparison of doses as a percent of $D_{\mathrm{Rx}}$ for egs_brachy ($D_{eb}$) and BrachyDose ($D_{BD}$) at points of interest. Statistical uncertainties are less than 0.5% for BrachyDose ^9^ and 0.08% for egs_brachy.

		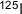		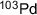	
Plaques	Points of interest	$D_{BD}$	$D_{eb}$	$D_{BD}$	$D_{eb}$
	Cornea	33.6	33.1	30.4	29.9
	Sclera	150	150	154	153
	Lens	61.1	60.4	61.0	60.5
Iris‐270	Eye center	24.4	24.0	21.0	20.5
	Macula	6.26	6.17	3.80	3.74
	Optic disk	6.56	6.45	4.05	3.96
	Cornea	72.6	72.2	74.0	73.7
	Sclera	154	154	162	161
	Lens	62.4	61.8	62.1	61.5
Iris‐270‐No‐Lips	Eye center	24.3	23.9	20.3	20.0
	Macula	6.39	6.30	3.76	3.70
	Optic disk	6.71	6.61	4.05	3.95
	Cornea	73.1	72.9	74.0	74.0
	Sclera	155	154	162	161
	Lens	62.2	61.6	61.7	61.4
COMS‐No‐Insert	Eye center	23.8	23.4	20.1	19.8
	Macula	6.14	6.00	3.68	3.62
	Optic disk	6.41	6.31	3.95	3.88
	Cornea	41.9	41.2	38.3	37.7
	Sclera	143	143	148	147
	Lens	79.3	78.5	80.7	79.8
Iris‐360	Eye center	31.4	30.9	27.4	26.8
	Macula	8.04	7.94	4.97	4.86
	Optic disk	8.43	8.37	5.30	5.23
	Cornea	25.5	25.1	22.5	22.2
	Sclera	161	160	164	163
	Lens	41.9	41.7	40.6	40.5
Iris‐180	Eye center	17.1	17.0	14.3	14.5
	Macula	4.41	4.45	2.60	2.67
	Optic disk	4.60	4.61	2.79	2.80

## 3 DATA FORMAT AND ACCESS

The iris plaque geometry models and an example egs_brachy input file are distributed alongside egs_brachy on GitHub, accessible at https://github.com/clrp‐code/egs_brachy. As with egs_brachy, the iris plaque geometric models are open‐source and thus may be freely accessed and used. Each plaque model has an individual folder with its respective geometry files and seed coordinates.

Dose distributions are distributed on Zenodo, the open source repository built and operated by CERN and OpenAire, and can be found online at https://doi.org/10.5281/zenodo.14776641. The dose distributions are organized into individual zipped folders for each plaque model presented in this work: Iris‐180, Iris‐270, Iris‐360, Iris‐270‐No‐Lips, and COMS‐No‐Insert. Each folder contains 3D dose distributions (.3ddose^35^) for 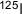, 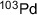, and 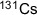 MC simulations. Those files present doses in terms of dose per unit seed air kerma strength (Gy 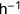 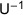). Additionally, the folder contains TG43‐sim dose distributions; note that TG43‐sim dose distributions are identical for Iris‐270, Iris‐270‐No‐Lips, and COMS‐No‐Insert because they have the same seed positions.

## 4 POTENTIAL IMPACT

The present work is the first time that iris plaque models for MC simulations have been developed and will be released with an open‐source MC code. It is also the first time that 3D dose distributions for iris plaques have been benchmarked and released. Overall, the egs_brachy dose distributions determined in this work are in agreement with previously‐published BrachyDose results for the iris plaques.^9^ The relatively small differences observed are consistent with observed discrepancies between egs_brachy and BrachyDose reported elsewhere, for example, for a COMS 16 mm plaque loaded with 6711 seeds,^14^ or in comparisons of various MC codes for eye plaque brachytherapy.^5^, ^16^, ^18^

The present work contributes to a growing collection of benchmarked MC plaque models and dosimetric datasets for ocular brachytherapy. Safigholi *et al* contributed eye plaque models for egs_brachy and associated 3D dose distributions for 10–24 mm COMS plaques (containing 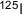 or 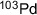 seeds), 12–22 mm Eckert & Ziegler BEBIG plaques (containing 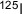 seeds), and four 16 mm diameter plaques representative of various models used worldwide (all in version 2 of the CLRP Eye Plaque Database (CLRP_EPv2)).^18^ Fletcher et al produced 3D dosimetric reference datasets for COMS eye plaque brachytherapy, with release of files from four MC codes (egs_brachy, MCNP6, Penelope2014, and TOPAS).^16^ The present work expands the scope of available MC plaque models and 3D dosimetric datasets to those used for iris melanoma brachytherapy. Thus, the present work supports advancement of ocular brachytherapy, addressing the recommendations of TG‐129,^10^ TG‐186^11^, and TG‐221^2^ by providing 3D dose distributions and open‐source models for use with egs_brachy to improve dose evaluations.

Eye plaque models developed in the present work will be distributed freely alongside egs_brachy. The egs_brachy files and 3D dosimetric datasets released are provided for research and educational purposes only and must not be directly used for clinical applications, unless explicitly recommended for clinical use by the appropriate professional body (e.g., AAPM). The files and datasets released may be useful for various activities. For example, the 3D dosimetric datasets may be useful to others in verifying their own MBDCA implementations for brachytherapy. The eye plaque models and input files may be used as a starting point for further calculations, for example, researchers could adapt the egs_brachy eye plaque models for simulations of different custom plaque models or use them with virtual patient models to develop custom patient‐specific treatment models.^36^, ^37^ Those engaged in learning about MBDCAs can advance their understanding of differences between TG‐43 and MC simulations by carrying out their own egs_brachy simulations and engaging in detailed analyses and comparison of 3D dose distributions.

## 5 CONCLUSION

Five plaque models used for iris melanoma brachytherapy have been successfully simulated in egs_brachy and benchmarked. The dose distributions along the central axis, at fixed radii in the eye, and at six points of interest were compared to previously published BrachyDose data. Overall, the dose distributions show agreement across all the plaques with discrepancies of few percent relative to BrachyDose data. The geometries of the plaques and their respective dose distributions are publicly available: eye plaque models will be distributed open source with egs_brachy and 3D dose distributions are available on Zenodo. It is hoped that these datasets will have uptake to advance medical physics research and practice globally.

## CONFLICT OF INTEREST STATEMENT

The authors declare no conflicts of interest.
